# Supplementary material for: Barriers and facilitators of advance care planning practices in multi-disciplinary, multi-facility palliative care for Japan’s aging population: A qualitative analysis
Source: PLoS One. 2025 May 28;20(5):e0323976. doi: 10.1371/journal.pone.0323976 (PMC12118854; doi:10.1371/journal.pone.0323976)
Supplement: S1 Appendix — (DOCX) [file pone.0323976.s001.docx]

**S1 Appendix. Understanding patients’ intentions**

| Barriers |  |
| --- | --- |
| 【Inability to understand the patient’s intentions】 |  |
| ―Many older community members face significant legal and ethical challenges before ACP can be initiated． | (B, E, O, U) |
| ~~―~~The patient’s true wishes remain unknown due to a lack of response to the professional’s engagement. | (J, P) |
| 【Not listening to the patient’s intentions】 |  |
| ―There is a prejudice that older adults with dementia cannot express their wishes. | (Q) |
| ―The conditions of the facility and the opinions of the family often take precedence over the patient’s wishes. | (P, Q, S, U) |
| Facilitators |  |
| 【Skills to understand patients’ intentions】 |  |
| ―Proficiency in inquiring about the wishes of seriously ill patients and understanding their intentions based on their conditions. | (C, D, G, M, P) |
| ―Obtaining information about the patient's wishes from family members and caregivers. | (E, O) |
| ―Discovering the patient’s wishes through progress records. | (D) |
| ―Capturing the essence of their wishes through various expressions and words. | (A, B, D, R, T) |
| 【Skills to better understand the true meaning of the patient’s intentions】 |  |
| ―Continuously reassessing and confirming the patient’s intentions as perceived by professionals. | (B, D, G) |
| ―Being present in the moment with the patient, determining the right time to ask about their willingness to move forward, and listening attentively. | (A, D, E, F, G, H, I, K, L, M, O, Q, R, S, T) |
