# Supplementary material for: Barriers and facilitators of advance care planning practices in multi-disciplinary, multi-facility palliative care for Japan’s aging population: A qualitative analysis
Source: PLoS One. 2025 May 28;20(5):e0323976. doi: 10.1371/journal.pone.0323976 (PMC12118854; doi:10.1371/journal.pone.0323976)
Supplement: S2 Appendix — (DOCX) [file pone.0323976.s002.docx]

**S2 Appendix. Family support**

| Barriers |  |
| --- | --- |
| 【Physical and psychological distance between patient and family】 |  |
| ―Difficulty in communicating directly with family members who live far away. | (B) |
| ―Challenges in building relationships with family members who only occasionally visit the facility. | (G) |
| 【Family’s lack of understanding and disagreement with the patient】 |  |
| ―Family members struggle to comprehend changes in the patient’s medical condition due to a lack of expectation that the patient will become seriously ill. | (G, K, Q, U) |
| ―Disagreements within the family arise from the patient’s transition to end-of-life care, shifts in family dynamics, and the patient bearing the burden alone. | (C, K, O, U, V) |
| 【Reduced family decision-making capacity】 |  |
| ―Both the patient and family members being older adults complicates decision-making, making it challenging to respect the patient’s wishes. | (L) |
| 【Insufficient care resources to support the family】 |  |
| ―Even if the person wishes to die at home, it is difficult without a support system that enables the family to provide care. | (U) |
| Facilitators |  |
| 【Early relationship building and discussions between professionals and families】 |  |
| ―When family members have differing opinions, finding a compromise by discussing the matter with the patient and all family members. | (M, Q, T) |
| ―Building relationships early, providing frequent information, and having discussions with family members help them adapt to changes in the patient’s condition. | (C, E, O, U) |
| 【Presuming the patient’s intentions and assisting the family in making decisions on the patient’s behalf】 |  |
| ―If the older patient’s wishes are not clear, informing the family about the advantages and disadvantages of treatment and encouraging them to think and discuss together. | (L) |
| ―In situations requiring a substitute decision-maker, ensure the family has enough information and time to understand the patient’s wishes. | (E, T) |
| 【Respecting the wishes of family members near the end of the patient’s life】 |  |
| ―Respecting the family’s wishes to prevent any regrets when the patient’s end-of-life is near. | (C, E, G, M, P, Q, U) |
| ―If it is difficult for the patient to complete the consent form for using the facility, having a family member complete it on their behalf. | (U) |
| ―Decisions about end-of-life care, including life-sustaining treatment for terminally ill patients, should also consider the wishes of the patient’s family. | (G) |
