# Supplementary material for: Barriers and facilitators of advance care planning practices in multi-disciplinary, multi-facility palliative care for Japan’s aging population: A qualitative analysis
Source: PLoS One. 2025 May 28;20(5):e0323976. doi: 10.1371/journal.pone.0323976 (PMC12118854; doi:10.1371/journal.pone.0323976)
Supplement: S3 Appendix — (DOCX) [file pone.0323976.s003.docx]

**S3 Appendix. Information sharing using tools**

| Barriers |  |
| --- | --- |
| 【Differing information needs and sharing methods at each facility】 |  |
| ―Checking the patient information sent, but responses are at the facility’s discretion. | (M) |
| ―The information received from other facilities may differ from the needs of the receiving facility. | (M) |
| 【Lack of recording skills】 |  |
| ―Inadequate descriptions capturing the main points of patient and family narratives. | (G) |
| ―Lack of understanding of proper record-keeping practices. | (D, J, Q, T) |
| 【Reluctance to widely share personal information】 |  |
| ―Concerns about the ethical implications of personal information flowing to external organizations. | (R) |
| Facilitators |  |
| 【Realization of daily care improvements through information sharing】 |  |
| ―A sense of smooth, personalized care tailored to the patient’s condition and situation. | (O, V) |
| ―Information sharing can help alleviate concerns about treatment and home care, which may otherwise be kept confidential. | (C, V) |
| 【Compatibility of daily tasks and ACP by utilizing tools】 |  |
| ―Creating records that are ACP-compatible as part of routine work. | (T) |
| 【Selecting the right tools suitable for individual cases and local conditions】 |  |
| ―Selecting the information-sharing tool best suited to the specific circumstances of each case. | (A, E, F, G, H, K, N, P) |
| ―Instead of strictly adhering to government guidelines, determining the best way to record information independently. | (V) |
| ―Improving methods of sharing patient information that align with the community’s characteristics through interdisciplinary discussion. | (N) |
| ―Adoption of cost effective tools for information sharing. | (V) |
| 【Evaluating and enhancing tools to improve the quality of ACP practice】 |  |
| ―Evaluating information sharing using tools from the perspective of the quality of care through ACP practice. | (H, O, T) |
| ―Improving recording forms so that ACP content is easily understood by different departments. | (O) |
