# Supplementary material for: Barriers and facilitators of advance care planning practices in multi-disciplinary, multi-facility palliative care for Japan’s aging population: A qualitative analysis
Source: PLoS One. 2025 May 28;20(5):e0323976. doi: 10.1371/journal.pone.0323976 (PMC12118854; doi:10.1371/journal.pone.0323976)
Supplement: S4 Appendix — (DOCX) [file pone.0323976.s004.docx]

**S4 Appendix. Collaboration among multiple professions**

| Barriers |  |
| --- | --- |
| 【Diverse behaviors and interpretations by patients/families depending on the profession】 |  |
| ―Different professions have varying interpretations of the wishes communicated by patients/families. | (C) |
| ―Different professions perceive and view patients differently. | (B, F) |
| 【Lack of understanding and indifference among other professionals toward ACP】 |  |
| ―Lack of understanding and interest in ACP among other professionals involved. | (D, E, P, T) |
| ―Information provided by one’s own profession is not utilized by multidisciplinary professionals. | (M) |
| Facilitators |  |
| 【Discussions grounded in solidarity among multidisciplinary colleagues】 |  |
| ―Discussing the patient’s condition, their words, and the opinions of multiple professionals, combining multiple perspectives. | (C, D, F) |
| ―Ease of communication is enhanced by face-to-face relationships among multiple professions. | (A, R) |
| 【Clarification of the role of each professional in practice】 |  |
| ―Clarification of the patient’s wishes through ACP helps establish a clear policy of care. | (G, I) |
| ―Dissemination of the role of one’s own profession in ACP implementation. | (F) |
