# Supplementary material for: Barriers and facilitators of advance care planning practices in multi-disciplinary, multi-facility palliative care for Japan’s aging population: A qualitative analysis
Source: PLoS One. 2025 May 28;20(5):e0323976. doi: 10.1371/journal.pone.0323976 (PMC12118854; doi:10.1371/journal.pone.0323976)
Supplement: S5 Appendix — (DOCX) [file pone.0323976.s005.docx]

**S5 Appendix. Cross-facility and cross-departmental cooperation**

| Barriers |  |
| --- | --- |
| 【Differences in required information and procedures under the medical and long-term care systems】 |  |
| ―Varied procedures between medical and long-term care systems require significant time and effort to synchronize. | (C) |
| ―Functional fragmentation between medical and nursing care in the community often result in discrepancies in information sharing. | (C, M) |
| ―Differences in operational practices among local facilities hinder mutual understanding and cooperation. | (U) |
| 【Limitations on patient acceptance criteria set by the facility】 |  |
| ―Facilities for older adults lacking end-of-life care experience may not accommodate patients approaching the end of life. | (U) |
| ―Facilities with policies against providing end-of-life care cannot offer such services even if patients express a desire for them. | (U) |
| 【Lack of information on ACP practices at other facilities】 |  |
| ―Uncertainty about the implementation status of ACP in other departments or professions leads to misunderstandings when sharing ACP information. | (Q, S) |
| ―Absence of administrative support for promoting ACP practices in the community. | (F) |
| Facilitators |  |
| 【Human connections between professionals in multiple facilities and departments】 |  |
| ―Face-to-face meetings and discussions among professionals from various departments and facilities facilitate effective communication about individual patient situations. | (B, C, D, G, H, I, K, L, M, O, R, S, V) |
| ―Presence of coordinators dedicated to integrating information from multiple professionals. | (J) |
| 【Communication skills to convey the patient’s wishes to multiple facilities】 |  |
| ―Obtaining family consent to share patient information across facilities. | (B) |
| ―For specific cases, conduct in-person communication to ensure clarity. | (B, K) |
| ―Communicate factual information and the patient’s expressed wishes in collaboration with other facilities. | (B) |
| ―Consistently convey the patient’s wishes regardless of the ACP implementation status of collaborating facilities. | (K, L) |
