# Supplementary material for: Barriers and facilitators of advance care planning practices in multi-disciplinary, multi-facility palliative care for Japan’s aging population: A qualitative analysis
Source: PLoS One. 2025 May 28;20(5):e0323976. doi: 10.1371/journal.pone.0323976 (PMC12118854; doi:10.1371/journal.pone.0323976)
Supplement: S6 Appendix — (DOCX) [file pone.0323976.s006.docx]

**6 Appendix. Raising community awareness**

| Barriers |  |
| --- | --- |
| 【Lack of end-of-life awareness among residents】 |  |
| ―Healthcare professionals are not actively informing citizens about end-of-life options. | (U) |
| ―Citizens lack a concrete understanding of their end-of-life choices. | (S, U) |
| 【Absence of a local culture to discuss death】 |  |
| ―The community lacks an atmosphere conducive to discussing death. | (H, L, S) |
| ―Citizens generally lack knowledge of ACP and tend to rely on doctors in case of illness. | (B, P, Q, S) |
| Facilitators |  |
| 【Raising awareness among residents and local professionals through daily work】 |  |
| ―Communicating ACP to collaborating facilities during patient transitions. | (C, M) |
| ―Extending ACP considerations from one's own facility and community role to encompass the entire citizenry. | (N, R, V) |
| 【Expression of willingness to engage in ACP from patients and families】 |  |
| ―When healthcare professionals suggest ACP to a patient’s family, it often leads to open discussions without resistance. | (A, F, Q) |
| ―Increasingly proactive inquiries from patients and families help initiate ACP conversations. | (F, L, S) |
