# Supplementary material for: Barriers and facilitators of advance care planning practices in multi-disciplinary, multi-facility palliative care for Japan’s aging population: A qualitative analysis
Source: PLoS One. 2025 May 28;20(5):e0323976. doi: 10.1371/journal.pone.0323976 (PMC12118854; doi:10.1371/journal.pone.0323976)
Supplement: S7 Appendix — (DOCX) [file pone.0323976.s007.docx]

**S7 Appendix. Efforts by implementation promoters and their departments**

| Barriers |  |
| --- | --- |
| 【A sense of burden and indifference among department staff】 |  |
| ―Staff feeling compelled to engage in ACP or displaying indifference. | (S) |
| ―Shortage of manpower and staff resistance due to complex and busy ward operations. | (N, Q) |
| 【Lack of ACP knowledge and skills among department staff, and care insecurity】 |  |
| ―Insufficient knowledge and skills of staff regarding ACP. | (D, G) |
| ―Concerns of caregivers about end-of-life care. | (C) |
| 【A sense of uncertainty about how to resolve ACP-related issues】 |  |
| ―Questioning the appropriateness of actions taken, making it challenging to enhance staff proficiency in ACP. | (A) |
| Facilitators |  |
| 【Engagement with ACP implementation】 |  |
| ―Professionals’ belief and values regarding ACP. | (B, K, L, M, O, P, Q, R, S, T) |
| ―Personal learning, satisfaction, and a sense of growth gained through ACP initiatives. | (A, C, D, E, G, I, J, M, O, P, Q, S, T) |
| 【Recognition of the effects of ACP activities within the organizational structure】 |  |
| ーIntegrating quality assurance practices into the foundational philosophy and structure of an organization through ACP initiatives. | (B, R, T) |
| ―Systematic ACP implementation has demonstrated positive impacts on quality of care. | (A, B, C, D, E, F, G, H, I, J, N, O, P, Q, R, S, T) |
| ―Encouraging staff participation in ACP by integrating it into daily operations. | (A, G) |
| 【Staff education within the department with emphasis on respect for the individual】 |  |
| ―Facilitating staff participation in ACP in an easily understandable manner. | (K) |
| ―Recognizing contributions each staff member can make, with varying degrees of interest and knowledge. | (S, T) |
| ―Preparing ACP materials that can be easily utilized in acute care wards. | (Q) |
| [ ACP initiatives driven by a sense of professional role within the organization] |  |
| ―Being aware of the role professionals can play based on their expertise and position with the ACP initiative. | (F, G, R, S) |
| ―Contributing to the multidisciplinary ACP team by utilizing the unique characteristics of one’s profession. | (R) |
| ―Utilizing ACP information in the execution of one’s daily work. | (H) |
