# Supplementary material for: Barriers and facilitators of advance care planning practices in multi-disciplinary, multi-facility palliative care for Japan’s aging population: A qualitative analysis
Source: PLoS One. 2025 May 28;20(5):e0323976. doi: 10.1371/journal.pone.0323976 (PMC12118854; doi:10.1371/journal.pone.0323976)
Supplement: Attachment — Submitted filename: Response_to_Reviewer.docx [file pone.0323976.s009.docx]

**Response to Reviewer’s Comments**

Thank you very much for reviewing our research paper. We appreciate your comments for further refinement. We have indicated below our response to your peer review comments.

1． **Remove the paragraph mentioning the participants' professions, as well as tables 1 and 2, including the population characteristics and their professional experience, from the "results part" and include them in the "study participants and recruitment part."**

Reply：

Thank you for your suggestion.

Paragraphs referring to participants' occupations, as well as Tables 1 and 2, which include population characteristics and their professional experience, are now removed from the “results part” and included in the “study participants and recruitment part”.

２． **In the survey method, more details are required, whether audio or visual recordings were used for data collection. Plus, were the observations or field notes described during or after the interview? Afterwards, were the transcripts returned to participants for correction or additional comments?**

Thank you for your comments.

The data information was enhanced. It was audio recorded and converted verbatim. I also added that the verbatim data was not returned to the study participants for correction or additional comments.

The timing of field notes has been expanded to include the following details: ‘During the interviews, any uncertainties in the background of the practices were recorded in field notes and confirmed with the research participants during the interviews. After the interviews, any uncertainties about the overall structure and status of the facility were noted in the field notes and confirmed with the research contact person. In this way, efforts were made to understand the facility's overall approach and state of practice.’

3． **Justify the chosen sample size by discussing the data saturation.**

Thank you for your suggestion.

We had not discussed data saturation. The participants in this study were limited by convenience sampling, so it is possible that we did not reach theoretical saturation. In this study, we interviewed people in various occupations in the local community, but we did not interview local social workers, local therapists, care managers, etc. Therefore, it is possible that other areas and factors will be extracted in the future. We thus added the following to the ‘Strengths, limitations, and future challenges’ section: ‘considering the diversity of the research participants, it is an objective of future research to confirm the validity of the results.’

4． **Indicate whether the participants provided any feedback on the findings.**

Reply：

Thank you for your helpful suggestion．

Regarding the analysis results, we have now stated that we discussed them with NO, who is familiar with the field context, but we did not provide feedback on the analysis results to all the research participants. Because there are issues regarding ensuring the data authenticity, in the ‘Strengths, limitations, and future challenges’ section, given that the analysis results were not provided as feedback to the research participants in this study, we have now added it will be necessary to confirm the validity of the results in future research.

As for other corrections, we have made changes to the way the doi is listed in the bibliography.

Ex.)

2.	Sano J, Hirazawa Y, Komamura K, Okamoto S. An overview of systems for providing integrated and comprehensive care for older people in Japan. Arch Public Health. 2023;81(1): 81. <https://doi.org/10.1186/s13690-023-01076-5>

　　　　　　↓

2.	Sano J, Hirazawa Y, Komamura K, Okamoto S. An overview of systems for providing integrated and comprehensive care for older people in Japan. Arch Public Health. 2023;81: 81. doi: 10.1186/s13690-023-01076-5.
