# Supplementary material for: Barriers and facilitators of advance care planning practices in multi-disciplinary, multi-facility palliative care for Japan’s aging population: A qualitative analysis
Source: PLoS One. 2025 May 28;20(5):e0323976. doi: 10.1371/journal.pone.0323976 (PMC12118854; doi:10.1371/journal.pone.0323976)
Supplement: Attachment — Submitted filename: Response_to_Reviewer_auresp_2.docx [file pone.0323976.s011.docx]

Response to Reviewer’s Comments

We would like to express our sincere gratitude for the important comments and suggestions you have provided and the opportunity to revise the paper.

1. Literature Review and Theoretical Framework:
   • Weakness: The literature review lacks depth, particularly in situating the study within the broader global context of ACP implementation.
   • Suggestion: Expand the review to include more international perspectives on ACP, particularly from comparable aging populations in Europe or North America. This will provide stronger context for the relevance of your findings.

Response:

Thank you for these valuable suggestions. In the introduction section, we have cited ACP research on aging populations in Europe and North America to provide an international perspective to this research. (Please see page 3).

2. Methods - Study Design:
   • Weakness: There is a lack of clarity in the recruitment process, particularly regarding how the participants were selected and whether any sampling biases were considered.
   • Suggestion: Provide more detailed justification for the sampling strategy, including any limitations posed by convenience sampling and whether theoretical saturation was achieved. Clarify how you ensured diversity in participants across facilities.

Response:

We have provided additional information regarding the sampling process.

We stated that participation was offered to 25 multi-professional members of the community-based palliative care team who attended the collaborative meetings between Hospital C and local healthcare facilities, and that 22 of them agreed to participate in the research (Please see page 7).

Regarding the suggested limitations, we have clarified that this study focused on pioneering initiatives and that the regional palliative care team consisted of individuals from various professions with a voluntary interest in palliative care; therefore, the study participants were a group with a high interest in ACP. We noted that professional diversity was insufficient, and theoretical saturation could not be confirmed (Please see pages 27–28).

3. Data Collection and Interview Process:
   • Weakness: Although the authors conducted semi-structured interviews, there is insufficient detail on how the interview guide was developed or validated.
   • Suggestion: Include a more comprehensive description of the interview guide development process. Did it undergo pilot testing? How were the questions aligned with the research aims?

Response:

We have responded to each of your suggestions as follows:

＞Include a more comprehensive description of the interview guide development process.

We have enhanced the description of the interview guide development process. Specifically, the results of the preliminary survey showed that the focus of ACP practice in community healthcare lay on the patients and their families, local residents, and multiple professions inside and outside of the facility. Thus, a foundation for regional collaboration was essential. Moreover, based on the preliminary survey, we stated that this study aimed to examine one region that had established a foundation for regional collaboration and identify the factors inhibiting or promoting ACP promotion. The interview guide was established based on these factors (Please see page 11–12).

＞Did it undergo pilot testing?

We have now noted that no pilot tests were conducted for this survey (Please see page 12).

＞How were the questions aligned with the research aims?

Based on the results of the previous survey, we defined the purpose of this survey as clarifying the details of ACP practice in the medical profession. We have strengthened the consistency between the research purpose and interview guide. (Please see pages 12, 22).

４．Analysis - Content Analysis:
• Weakness: The data analysis section lacks transparency, particularly in the coding process and how inter-rater reliability was assessed.
• Suggestion: Provide more information on the coding process, including how discrepancies were resolved between researchers. Additionally, discuss the use of NVivo software and whether a formal inter-coder reliability measure was calculated.

Response:

Thank you for your comments. The analysis for this study reached a consensus among the researchers using NVIVO functions. We have now added the following details (Please see page 13).

-A description of the analysis process by the two researchers.

-Any disagreements during the analysis were addressed using specific examples, resolved by returning to the verbatim record and discussions employing the NVIVO function.

-The survey followed a consensus-based process, and inter-rater reliability was not measured.

-We have noted in the limitations and implications section that further survey research is necessary.

1. Results Presentation:
   • Weakness: The results section is overwhelming due to the sheer volume of data presented in tables and appendices, which impedes clarity.
   • Suggestion: Simplify the presentation of key barriers and facilitators in the main text. The tables could be reduced or moved to supplementary material. Focus more on high-level findings in the main results section.

Response:

We appreciate your valid comments regarding the duplication of information on the barriers and facilitators. To make the results section more concise, we have made the following changes (Please see pages 14–21).

- The barriers and facilitators were only included in the main text.

- They were deleted from the table, and only the list of domains was included.

- The sections’ focus was shifted to the main results; the barriers and facilitators and the quotations were deleted from the main text.

- The barriers that did not produce important results were deleted from the Abstract.

You mention simplifying the appendices; however, we have left them unchanged to ensure that practical details regarding the barriers and facilitators can be verified.

1. Discussion and Implications:
   • Weakness: The discussion lacks critical reflection on the practical implications of the findings for policy and practice.
   • Suggestion: Expand the discussion to include a more robust analysis of how these findings could inform future ACP practices in Japan. Consider discussing potential interventions or policy changes to address the barriers identified

Response:

We have reviewed and incorporated new literature to foster critical reflection on policies, practices, and intervention measures based on our findings (Please see page　25- 26).

-Our discussion emphasizes the skills required to discern the true intentions of older adults, as highlighted in the results. Considering Japanese policies and the cultural characteristics of relational autonomy in Asia, we highlighted the importance of developing the capacity of professionals to serve effectively as Advocates.

-Based on the above revisions, we have also revised the conclusion and abstract.

７．Limitations:
• Weakness: The limitations section is too brief and underplays key methodological concerns.
• Suggestion: Acknowledge more explicitly the limitations of convenience sampling, single-point data collection, and the exclusion of certain stakeholders such as social workers and therapists. Reflect on how these factors may have affected the findings

Response:

Thank you for your comments. These will aid our future research. We have made the following changes to these sections (Please see pages 27–28).

-The study had limitations. The study population did not reach theoretical saturation owing to the use of convenience sampling from the members of community palliative care teams in a limited number of communities. Specifically, the study did not include professionals who coordinate long-term care in the community, such as community social workers and care managers.

-Owing to these limitations, our study did not identify differences in the barriers and facilitators experienced by various professions involved in ACP implementation. Barriers and facilitators related to the economic challenges faced by older adults (e.g., poverty) and those reflecting the impact of broader social structures, including a declining birthrate and aging population, were not extracted. The perspectives of professionals who provide long-term support focusing on the declining physical condition of older adults were also not included. We acknowledge the limitations in the comprehensiveness of ACP implementation content addressed by local multi-facility and multi-professional groups.

-Regarding the survey’s cross-sectional nature, it did not capture the back-and-forth relationship between the barriers and facilitators in practice or the intervening factors. Additionally, the study did not clarify the background factors underlying each of the barriers and facilitators.

1. Language and Clarity:

• Weakness: The manuscript suffers from occasional awkward phrasing and unclear sentence structure, which hinders readability.
• Suggestion: A thorough language edit is needed to improve clarity and flow, particularly in the abstract and results sections.

Response:

Thank you for your suggestion. Regarding the paper’s language, we have again thoroughly reviewed for awkward expressions and unclear sentence structures. We have improved clarity and flow, particularly in the Abstract and Results sections (Please see page 2, 14-21). We have also used an academic editing service, and a proofreading certificate is attached for your review.
