# Supplementary material for: Barriers and facilitators of advance care planning practices in multi-disciplinary, multi-facility palliative care for Japan’s aging population: A qualitative analysis
Source: PLoS One. 2025 May 28;20(5):e0323976. doi: 10.1371/journal.pone.0323976 (PMC12118854; doi:10.1371/journal.pone.0323976)
Supplement: Attachment — Submitted filename: Reviewercomments .docx [file pone.0323976.s010.docx]

Title: **Barriers and facilitators of advance care planning practices in multi-disciplinary, multi-facility palliative care for Japan’s aging population: A** qualitative analysis

**Dear respectful Editor**

**Thank you for your invitation to review** the revised version of the manuscript" *Barriers and facilitators of advance care planning practices in multi-disciplinary, multi-facility palliative care for Japan’s aging population: A qualitative analysis* **"**, and I am pleased to note that the authors provide valuable insights into the challenges and enablers of advance care planning for aging population . The overall quality of the manuscript is good. I believe the manuscript is suitable for publication after minor revisions.

**Dear Authors,**

I would like to appreciate your effort to address such highly significant and essential concepts related to advanced care to aging population. The manuscript is well-structured, methodologically sound, and contributes to an important field of research, particularly in the context of Japan’s aging population; but I want to add some suggestions for feedback

- Although the research addresses a critical issue in palliative care, particularly in Japan, where an aging population necessitates effective ACP strategies. but still some terms as (e.g., community-based palliative care) need to emphasize how their practical implementation in Japan versus other countries.
- The manuscript presents an extensive list of barriers and facilitators but it still lacking how to link these factors more explicitly to broader healthcare policies and previous research findings
- More in-depth exploration of potential solutions to lessen the barriers would strengthen the paper.
- Addressing how societal attitudes towards shape ACP acceptance would provide more understanding
- Few grammatical inconsistences
