# Supplementary material for: Barriers and facilitators of advance care planning practices in multi-disciplinary, multi-facility palliative care for Japan’s aging population: A qualitative analysis
Source: PLoS One. 2025 May 28;20(5):e0323976. doi: 10.1371/journal.pone.0323976 (PMC12118854; doi:10.1371/journal.pone.0323976)
Supplement: Attachment — Submitted filename: Response_to_Reviewer_auresp_3.docx [file pone.0323976.s012.docx]

Response to Reviewer’s Comments

We would like to express our sincere gratitude for the insightful comments and suggestions you provided and the opportunity to revise the paper.

1. Although the research addresses a critical issue in palliative care, particularly in Japan, where an aging population necessitates effective ACP strategies. but still some terms as (e.g., community-based palliative care) need to emphasize how their practical implementation in Japan versus other countries.

Response:

The following supplementary explanations have now been added to the paper’s definitions of ACP and CBPC.

・We enhanced the discussion of ACP’s definition, explaining that we have chosen a definition that is suitable for Japan's aging population.

*There are several definitions of ACP[2,29,34]. In this study, we adopted that of the Japan Geriatrics Society, given the reality of Japan's aging population: “ACP is a process that supports people in making decisions about their future medical and long-term care, respecting each individual as a human being.”* *(Please see page 6).*

・Following Reviewer #4's suggestion, we have added a definition that includes a practical method reflecting the distinct attributes of Japan's healthcare delivery system.

*Community-based palliative care (CBPC) is an emerging field that seeks to integrate palliative and serious illness care with local health care systems[36]. Since Japan has a universal health insurance system and local residents can use any medical facility in the community, the definition included the practice method of multi-professional collaboration in multiple facilities. We define CBPC as palliative care provided by multidisciplinary healthcare professionals from multiple hospitals and community facilities who work together to deliver optimal care for patients and families who select and use multiple facilities from the final phase of life until death. (Please see page 7).*

1. The manuscript presents an extensive list of barriers and facilitators but it still lacking how to link these factors more explicitly to broader healthcare policies and previous research findings

Response:

As a result of this study, barriers and facilitators in seven practice domains were identified. These items were obtained in areas where a regional collaborative network had been established. Previous studies have also clarified that a regional collaborative network is necessary for improving the quality of regional palliative care. Based on these findings, we added the following to the “Strengths, limitations, and future challenges” section: the importance of a regional collaborative network for the implementation of ACP in a community. (Please see pages 28).

Reviewer #4's second suggestion relates closely to Reviewer #4's third suggestion. Please also see our response below.

1. More in-depth exploration of potential solutions to lessen the barriers would strengthen the paper.

Response:

We deepened our discussion of the barriers and facilitating factors in the seven practice domains, citing relevant literature, and identified three potential solutions. We have added this to the “Strengths, limitations, and future challenges” section. The added text is as follows. (Please see page 28)

*Despite the above limitations, this study suggests potential solutions for advancing ACP implementation in complex communities. First, the barriers and facilitators across the seven practice areas should inform the consideration and planning of ACP interventions, taking into account the current situation and the daily realities of community practice. This is particularly important when ACP is implemented in a community where multiple professionals collaborate as part of a CBPC team. Second, since previous studies have shown that a regional collaborative infrastructure improves the quality of palliative care in the community [58], this study’s results indicate that building a regional collaborative network system should be a priority, and CBPC have shown evidence of improved quality of care and reduced costs[32,59], CBPC teams should function at the top of the regional collaborative system. Third, barriers to and facilitators of “Understanding patients’ intentions,” which reflect the process of understanding others, include communication skills that account for the characteristics of older adults who become frail and experience cognitive decline as they approach the end of life. Additionally, the cultural values of the East Asian region which prioritize relationships, play a significant role in this process [56]. In a society where the number of elderly people will continue to increase, the communication skills of ACP professionals should be improved by considering relational autonomy. In addition, as a priority, the individual should be supported in selecting a trusted other as a proxy. This may be the best approach to achieve respect for personal autonomy based on trust and mutual understanding, which is acceptable to people's sociocultural attitudes.*

1. Addressing how societal attitudes towards shape ACP acceptance would provide more understanding

Response:

Thank you for your valuable comment. From the perspective of relational autonomy found in previous research, we have sought to gain a deeper understanding of the results and identify a direction that is more in line with social attitudes, offering a solution to potential challenges.

Reviewer #4's fourth comment also relates closely related to Reviewer #4's third comment. Please also see our third response, above. (Please see page 28)

５． Few grammatical inconsistences

Response:

We have reviewed the manuscript again, thoroughly proofread the English, and made necessary corrections. We have also revised the grammatical inconsistences and highlighted these revisions.

1. Other corrections

・In line with the above corrections, we have changed the term “regional information sharing system” used in the Abstract to “regional collaborative network system” to ensure consistency. (Please see page 2)

・Duplicate sentences in the “Strengths, limitations, and future challenges” section have been deleted. (Please see page 27)

・The reference list has been updated and the numbering has been corrected. (Please see page30-)
